# An alpha-helix variant p.Arg156Pro in LMNA as a cause of hereditary dilated cardiomyopathy: genetics and bioinfomatics exploration

**DOI:** 10.1186/s12920-023-01661-1

**Published:** 2023-10-02

**Authors:** Lei Chang, Rong Huang, Jianzhou Chen, Guannan Li, Guangfei Shi, Biao Xu, Lian Wang

**Affiliations:** 1 Department of Cardiology, Nanjing Drum Tower Hospital, Clinical College of Nanjing Medical University, Nanjing, Jiangsu 210008 China; 2 Department of Cardiology, Suzhou Dushu Lake Hospital, Dushu Lake Hospital Affiliated to Soochow University, Medical Center of Soochow University, Suzhou, 215000 China; 3 Department of Cardiology, Nanjing Drum Tower Hospital, Nanjing University Medical School, Nanjing, Jiangsu 210008 China

**Keywords:** LMNA, Nuclear lamina, Dilated cardiomyopathy, Alphafold2, Alpha-helix, Bioinformatics analysis

## Abstract

*LMNA* gene encodes lamin A/C protein which participates in the construction of nuclear lamina, the mutations of *LMNA* result in a wide variety of diseases known as laminopathies. LMNA-related dilated cardiomyopathy(LMNA-DCM) is one of the more common laminopathy which characterized by progressive heart failure and arrhythmia. However, the mutation features of LMNA-DCM are yet to be elucidated. Herein we described a dilated cardiomyopathy family carrying novel variant c.467G > C(p.Arg156Pro) of LMNA as heterozygous pathogenic variant identified by whole-exome sequencing. With the help of Alphafold2, we predicted mutant protein structure and found an interrupted α-helix region in lamin A/C. In the analysis of 49 confirmed pathogenic missense of laminopathies, Chi-square test showed the DCM phenotype was related to the α-helix region mutation (p < 0.017). After screening the differentially expressed genes (DEGs) in both mice models and human patients in Gene Expression Omnibus database, we found the variation of α-helix-coding region in LMNA caused abnormal transcriptomic features in cell migration, collagen-containing extracellular matrix, and PI3K-Akt signaling pathway. Subsequently we constructed (TF)-mRNA-microRNA (miRNA) regulatory network and identified 7 key genes (*FMOD*, *CYP1B1*, *CA3*, *F2RL1*, *HAPLIN1*, *SNAP91*, and *KANSL1*) as potential biomarkers or therapeutic targets in LMNA-DCM patients.

**Supplementary Information:**

The online version contains supplementary material available at 10.1186/s12920-023-01661-1.

## Introduction

**Fig. 1:**
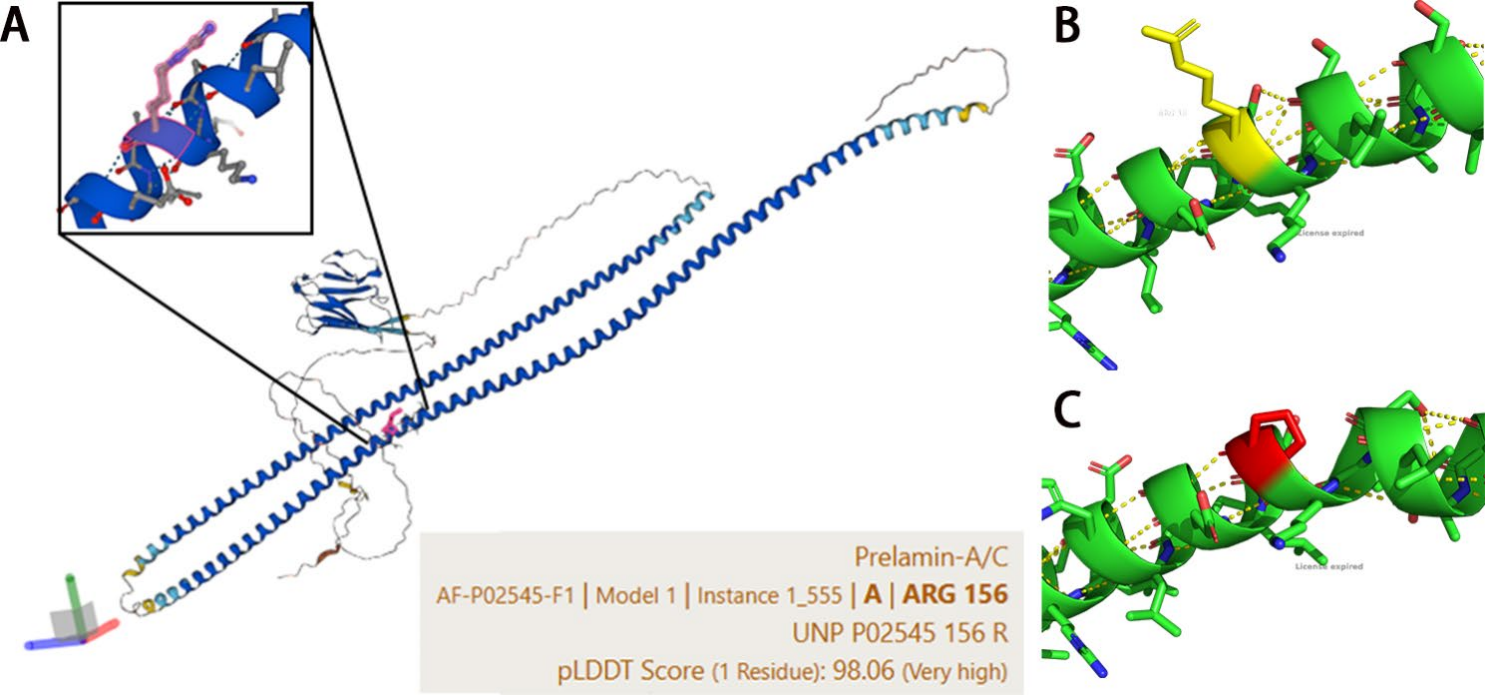
**(A)** The structure of lamin A/C. **(B)** The wild type α-Helix structure in object region. **(C)** The pathongenic α-Helix variant of lamin A/C-p.Arg156Pro(*LMNA*-c.467G > C)

*LMNA* gene encodes nuclear lamin A and lamin C (hereafter lamin A/C) which participate in the construction of nuclear lamina. Lamin A/C proteins have key roles in nuclear structural integrity and chromosomal stability. The mutations in *LMNA* could cause various diseases which called laminopathies, such as LMNA-related dilated cardiomyopathy (LMNA-DCM), Emery-Dreifuss muscular dystrophy (EDMD), lipodystrophy, skeletal dysplasia, and Hutchinson-Gilford progeria syndrome (HGPS) [1]. Different laminopathies show different tissues involvement and different clinical symptoms. Among these, LMNA-DCM is characterized by heterogeneous clinical manifestations, such as predominant structural heart disease, global myocardial dysfunction, conduction system abnormalities, and life-threatening arrhythmias. In some patients, sudden cardiac death (SCD) could be first manifestation. Mutations in *LMNA* account for 10% of hereditary DCM and up to 33% atrioventricular conduction disorders in DCM [2]. *LMNA* gene encodes lamin A/C which is widely expressed in various cells. In addition to the structural and supportive function in the nucleus, lamin A/C participates the regulation of gene expression through interaction with transcription factors (TFs), DNA, and chromatin [3]. Dhe-Paganon et al. firstly reported the three-dimensional (3D) crystal structure of lamin A/C globular tail through X-ray diffraction [4]. An in-depth study of the protein structure revealed that lamin A/C is composed of two α-helical chain domains and a globular immunoglobulin (Ig)-like domain containing several antiparallel β-strands [Fig. 1A]. Moreover, α-helix domain is highly conserved and critical for the formation of the α-helical coiled-coil dimer, which is ascribed as the basic building block for the construction of lamin filaments. It is reported that the level of mutated lamin A/C proteins was correlated with the severity of LMNA-DCM [5]. Hence, the structure variation study of lamin A/C could provide novel insight into the pathogenic process in LMNA-DCM.

LMNA-DCM is highly penetrant, adult-onset, malignant, and autosomal dominant, which is one of the most aggressive and lethal heart diseases [6, 7]. The clinical management strategies for LMNA-DCM are identical to other cardiomyopathies or heart failure. These approaches include symptomatic and supportive treatment with pharmacologic (neurohormonal antagonists, diuretics for congestion, and vasodilators for hemodynamic unloading) and ventricular device therapies. Prospective studies have shown that life-threatening ventricular arrhythmias are common in LMNA-DCM patients with LMNA mutations and significant cardiac conduction disorders, even if left ventricular ejection fraction (LVEF) is preserved [8]. Hence, the prophylactic implantable cardioverter-defibrillator (ICD) or cardiac resynchronization therapy implantation is recommended in this patient population [9]. Nonetheless, the current treatment strategy is inadequate for reversing the poor prognosis of LMNA-DCM. The exploration of novel therapies is the hot spot in LMNA-DCM research. Parisha et al. demonstrated that the human induced pluripotent stem cells derived cardiomyocytes from LMNA-DCM patients have aberrant nuclear morphology and specific disruptions in the peripheral chromatin [10]. Wei et al. utilized a mouse model and demonstrated the abnormal activation of the extracellular signal-regulated kinase (ERK) and the c-jun N-terminal kinase (JNK) branches [11]. Bioinformatic analysis found that the abnormal binding between lamin A/C and euchromatin DNA and the dysregulation of WNT/β-catenin or TGFβ-BMP pathways might be the putative molecular pathogenesis mechanism underlying LMNA-DCM [12]. Clinical trials have shown that lovastatin improves endothelial dysfunction and cellular crosstalk in LMNA cardiomyopathy [13]. In the mouse model, histone demethylase LSD1 and BET bromodomain inhibitor seem to be the therapeutic targets for preventing or curing LMNA-DCM [14, 15].

Despite the recent advances in clinical and molecular studies, a specific treatment for LMNA-DCM is yet lacking [16]. Herein, we investigated a family with LMNA-DCM and analyzed the genotype–phenotype correlation of 49 LMNA pathogenic variants. Protein structure prediction and bioinformatics analysis summarized the special structural variants in LMNA-DCM. Transcriptome analysis on specific LMNA mutant mice models and patient samples provided a new perspective for pathogenesis and a potential therapeutic direction in LMNA-DCM.

## Methods

### Objectives and clinical evaluation

A total of 8 individuals (4 males and 4 females) across three generations from a Chinese pedigree with DCM were enrolled in the study in the Department of Cardiology, Nanjing Drum Tower Hospital in 2021. The proband is a 42-year-old woman who suffered from lower extremity edema and shortness of breath. She was also diagnosed with DCM by echocardiography and cardiac magnetic resonance. The clinical data of all family members, including clinical symptoms, physical examination, electrocardiogram, and echocardiography were collected for clinical evaluation. All the participants signed the informed consent form. This study was in accordance with the 1964 Helsinki Declaration and the ethical guidelines of the local ethics committee at the Affiliated Drum Tower Hospital.

### Whole-exome sequencing and analysis of gene variant

A volume of 10 mL of peripheral blood was collected from the proband and each of the seven relatives and stored at − 80°C. Whole exome sequencing (WES) and data analysis were conducted through BGI Co, Ltd (Shenzhen, China). gDNA was extracted according to the manufacturer’s standard procedure (MagPure Buffy Coat DNA Midi KF Kit). gDNA (Quality Control (QC): gDNA concentration ≥ 20 ng/ul, total DNA ≥ 1000 ng) was broken into 100 to 500 bp fragments by BGI’s enzyme kit (Segmentase, BGI). The 180 to 280 bp fragments were then collected by magnetic bead. The collection added ‘A’ base at 3’ overhangs after repairing ending, which made sure the fragments could pare ‘T’ base with special adapter, and built single individual DNA library after ligation-mediated PCR and purification (QC: small fragment concentration ≥ 40 ng/ul, total gDNA ≥ 1000 ng). The exon-coding region of the sample gene and its upstream and downstream 20 bp regions were captured and amplified using a Roche KAPA HyperExome chip. After quality inspection, the MGISEQ-2000 high-throughput sequencing platform of MGI was used for sequencing [17]. The average depth of the sample sequencing was 180X, and the sequencing depth of the core target sequence region was ≥ 20X. The region of the gene variant was amplified by polymerase chain reaction (PCR) in the proband and seven relatives. The PCR products were used to verify the gene variant by Sanger sequencing. Then, ESP6500 (http://evs.gs.washington.edu/EVS/), gnomAD (http://gnomad.broadinstitute.org/), HGMD (http://www.hgmd.org), and OMIM databases (http://www.omim.org) were used to annotate the data after filtering. The pathogenicity of the candidate gene variant sites was predicted by SIFT (http://sift.jcvi.org), MutationTaster (http://www.mutationtaster.org/), and Condel (https://sourceforge.net/projects/condel/). The genetic variant detected was explained according to the guidelines of the genetic variant classification standard of the American College of Medical Genetics and Genomics.

### Protein conformational analysis and prediction

The protein sequence of the wild-type (WT) *LMNA* gene (UniProt Protein Symbol: P02545) was downloaded from the UniProt database (https://www.uniprot.org/). The variant protein sequence was edited based on the WT protein sequence. AlphaFold2 with a template in CoLab (https://colab.research.google.com/github/sokrypton/ColabFold/blob/main/AlphaFold2.ipynb#scrollTo=UGUBLzB3C6WN) was used to construct the conformation of the WT and variant protein. Pymol 2.5 was used to visualize the various conformations of the proteins [18].

### Phenotype-gene data of LMNA pathogenic mutations and statistical analysis

Firstly we downloaded 58 pathogenic allelic variants of *LMNA* mutation from the Online Mendelian Inheritance in Man (OMIM) database (http://www.omim.org). To explore the effect of the certain mutation location on the phenotype, we only selected 48 missense mutations for the subsequent study, while 10 nonsense, frameshift, and inframe mutations were excluded. And then we rechecked the phenotype of above 48 mutations in the PubMed database and finally identified 36 missense mutation locations which possess clear genotypic and phenotypic relationship. At the same time We retrieved the PubMed databases and added 12 missense mutation locations coming from retrospective study and cohort study. Combined with new mutation location in our family study, totally 49 confirmed pathogenic missense mutation locations of *LMNA* and the related phenotype information were included in this study [Supplementary Material 1]. Every mutation was grouped either into the cardiomyopathy or the non-cardiomyopathy group according to the phenotype. Based on the secondary and tertiary structure of lamin A/C, the amino acid sequence was divided into different segments, which consisted of a composite chain structure (amino acids 1–386), a globular immunoglobulin (Ig)-like domain (amino acids 427–547), and a single chain (amino acid 548–661). The composite chain structure consists of three single chains and two α-helixes (amino acids 26–226 and amino acids 231–386). The statistical analysis of the enumeration data was performed using SPSS for Windows (SPSS, Chicago, IL, USA). The significance of the associations was defined using Pearson’s chi-square test.

### Transcriptome data source

All the microarray and high-throughput sequencing datasets were downloaded from the Gene Expression Omnibus (GEO) database (http://www.ncbi.nlm.nih.gov/geo). LMNA and DCM were used as keywords to search for gene-expression datasets with respect to mice or human heart tissue. Finally, six datasets were selected from GEO. GSE123916 (4 LMNA-DCM models and 4 normal controls) is an RNA-sequence dataset that includes LMNA-D300N mutant and WT control heart tissues from 2-week-old mice hearts. GSE36502 (3 LMNA-DCM models and 3 normal controls) and GSE6398 (6 LMNA-DCM models and 8 normal controls) were microarray datasets obtained from 6-week-old LMNA-N195K mutant mice, 10-week-old LMNA-H222P mutant mice, and controls. GSE17476 (2 LMNA-DCM patients and 2 normal controls) was compiled from LMNA-E161K mutant DCM human family. The above mutations were all present in the α-helix region of lamin A/C. GSE146621 (7 LMNA-DCM patients and 22 other hereditary DCM) was a DCM sequencing dataset obtained from various pathogenic genes such as *LMNA*, *MYH7*, *RBM20*, and *TTN*. GSE120836 (5 LMNA-DCM patients and 5 normal controls) was a sequencing dataset from LMNA-DCM and used for the validation set in subsequent analyses.

### Identification and enrichment analyses of DEGs

The microarray datasets (GSE36502, GSE6398, and GSE17476) were analyzed by GEO2R to identify DEGs. The DEGs of high-throughput sequencing datasets GSE123916 and GSE146621 were identified using the edgeR analysis package in R statistical program. All the DEGs were defined as genes with the significance level set at q < 0.05 and |log fold-change|>1.5. The Venn diagram was constructed to detect and visualize the overlapping DEGs of the LMNA-DCM mice model datasets. Metascape [19] (https://metascape.org) was used to conduct the Gene Ontology (GO) enrichment and Kyoto Encyclopedia of Genes and Genomes (KEGG) pathway [20] analysis to elucidate the biological characteristics of the objective DEGs list. The mice model overlapping DEGs (at least differentially expressed in two datasets) was firstly analyzed in Metascape. The DCM patient DEGs were divided into up and downregulated DEGs for subsequent analysis. Finally, the DEGs of GSE146621 were enriched to clarify the specific characteristics of LMNA-DCM compared to other hereditary DCM.

### Selection of key genes

**Fig. 2:**
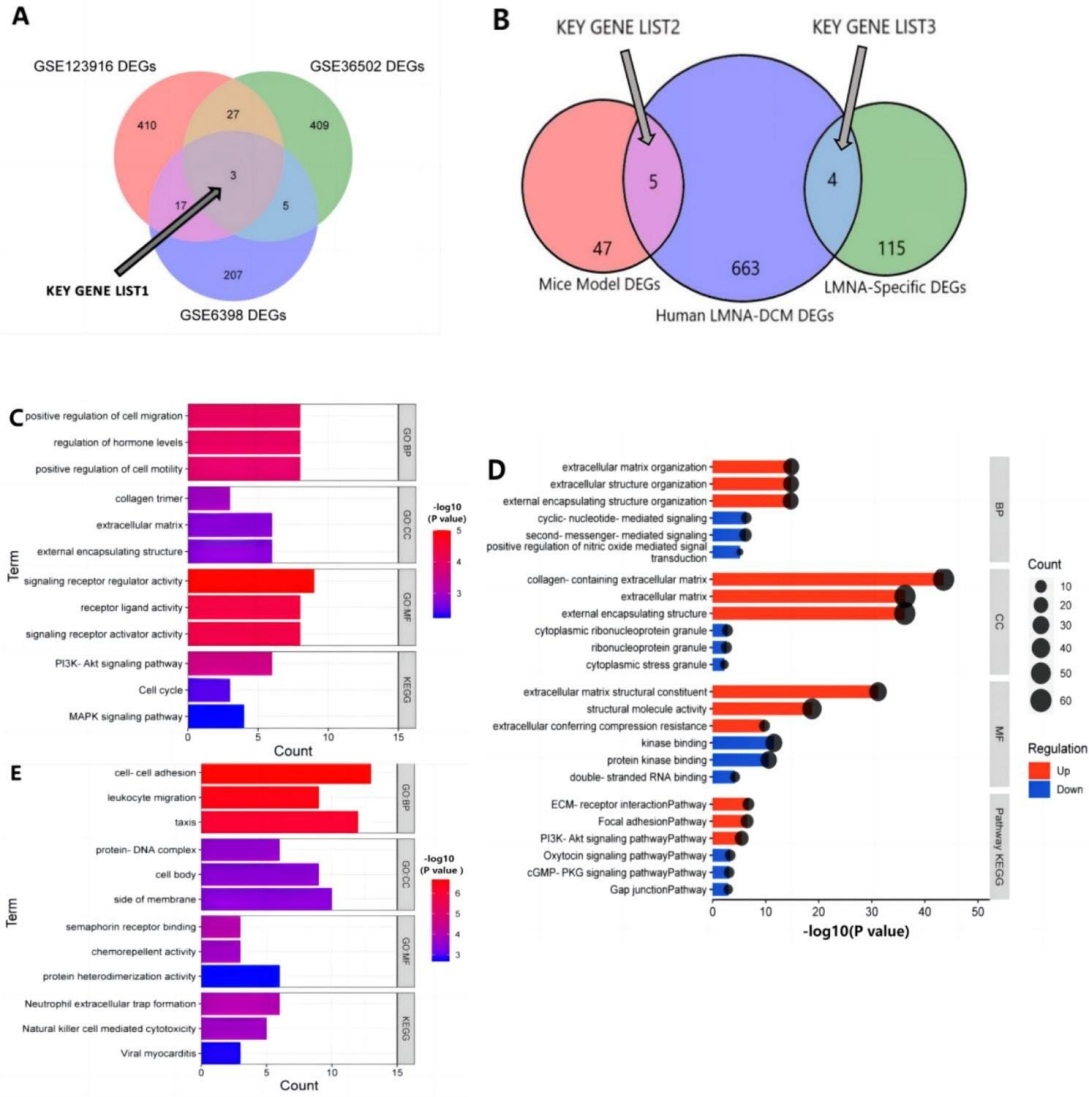
**(A)**Venn diagram depicted the DEGs by the 3 Mice cohorts. 3 of 52 common DEGs were identified as Key Gene List1. **(B)** Venn diagram showed the intersection between Mice Model common DEGs, Human LMNA-DCM DEGs and LMNA-specific DEGs. The overlapping DEGs were identified as Key Gene List2 and List3. **(C)** The enrichment analysis of the 52 Mice Model common DEGs by Metascape. **(D)** Analysis of function and enrichment of Human LMNA-DCM DEGs. The enriched items of up-regulated genes were red, while the enriched items of down-regulated genes were blue. **(E)**The enrichment analysis of the 119 LMNA-specific DEGs

The key genes were deemed vital in the pathogenic process and as potential therapeutic targets in LMNA-DCM. Herein, we adopted three methods to screen the key genes. In mice cohorts, the common DEGs were obtained by the intersection of the genes between at least two cohorts. And the DEGs overlapping with the 3 mice cohorts were classified into the key gene list 1 [Fig. 2A]. The human LMNA-DCM DEGs were identified between LMNA-DCM patients and healthy controls. While the human LMNA-DCM DEGs also detected in at least two mice cohorts, these DEGs were classified as key gene list 2. In order to explore the specific transcriptome modifications between LMNA-DCM and other hereditary DCM, we identified LMNA specific DEGs between LMNA-DCM patients and other hereditary DCM patients. The key gene list 3 was constituted by the overlapping genes between LMNA-DCM DEGs and LMNA specific DEGs [Fig. 2B].

### TF-mRNA-microRNA (miRNA) regulatory network construction and validation of the key genes

Mirwalk [21], a miRNA-target interactions database, was utilized to predict the miRNAs interacting with the key genes. The transcriptional Regulatory Relationships Unraveled by Sentence-based Text Mining (TRRUST) database [22] was applied to predict the regulatory correlations between key genes and TFs. Subsequently, the TF-mRNA-miRNA regulatory network was integrated and visualized by Cytoscape soft [23]. Finally, the expressions of the key gene were verified in GSE120836 (5 patients with LMNA-DCM and 5 controls). A comparison of the two datasets was performed using the t-test. p < 0.05 was considered statistically significant.

## Results

### Clinical data analysis of pedigree

**Fig. 3:**
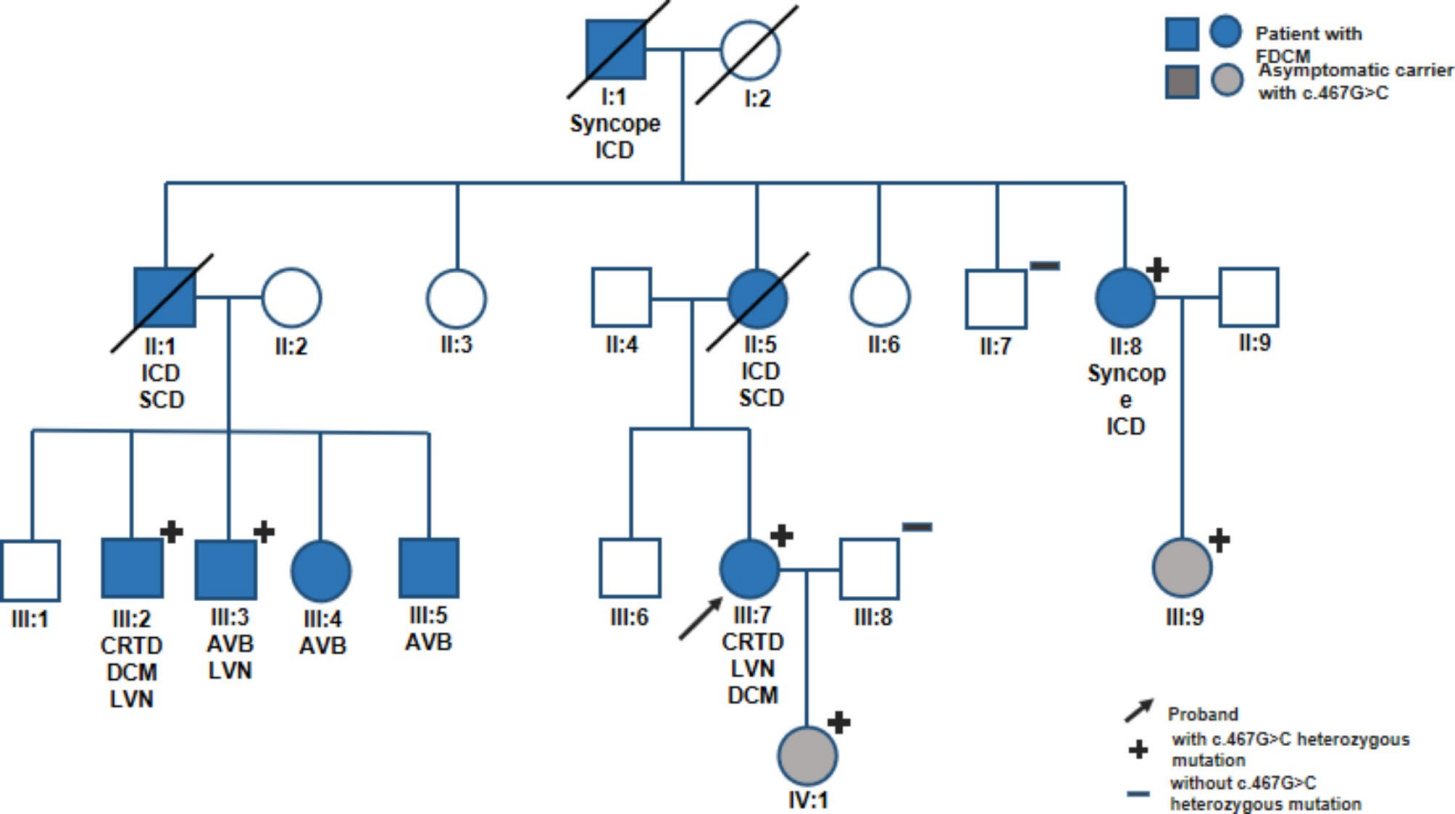
Pedigree of the Chinese family with inherited dilated cardiomyopathy. Females are depicted as circles and males as squares. Blue solid squares and circles denote suffering from DCM. Squares and circles with a crossing-slash indicate deceased individual, asterisks denote family members whose samples were sequenced and proband indicated with an arrow. Genotypes for LMNA were illustrated below the icons for individual with a plus (+) indicating c.467G > C heterozygous mutant and a minus (–) indicating wildtype for that genotype. The gray squares and cycles mean asymptomatic carrier with LMNA-c.467G > C

The pedigree of the familial DCM is shown in Fig. 3. The proband (III:7) visited our hospital at the age of 42 with orthopnea and lower extremity edema. Echocardiography revealed significantly reduced LVEF (30%) with enlargement of the left ventricle and left ventricular noncompaction (LVNC). Electrocardiogram showed high-grade atrioventricular block and frequent ventricular premature beat. After 3 months of heart failure drug therapy and reassessment, the patient received cardiac resynchronization therapy with defibrillator (CRT-D) implantation and a close follow-up. Her grandfather (I:1), mother (II:5), uncle (II:1), and aunt (II:8) received implantable cardioverter-defibrillator implantation for cardiogenic syncope. However, her mother (II:5) and uncle (II:1) died of sudden cardiac death. Her cousins (III:2, III:3, III:4, and III:5) suffered from various degrees of palpitations and shortness of breath. Their electrocardiogram showed type II atrioventricular block. Moreover, III:2 had the most severe heart failure symptom and received CRT-D implantation for echocardiography, which revealed distinct LVNC and dilated heart (EF 25%).

### Identification of pathogenic gene mutations

**Fig. 4:**
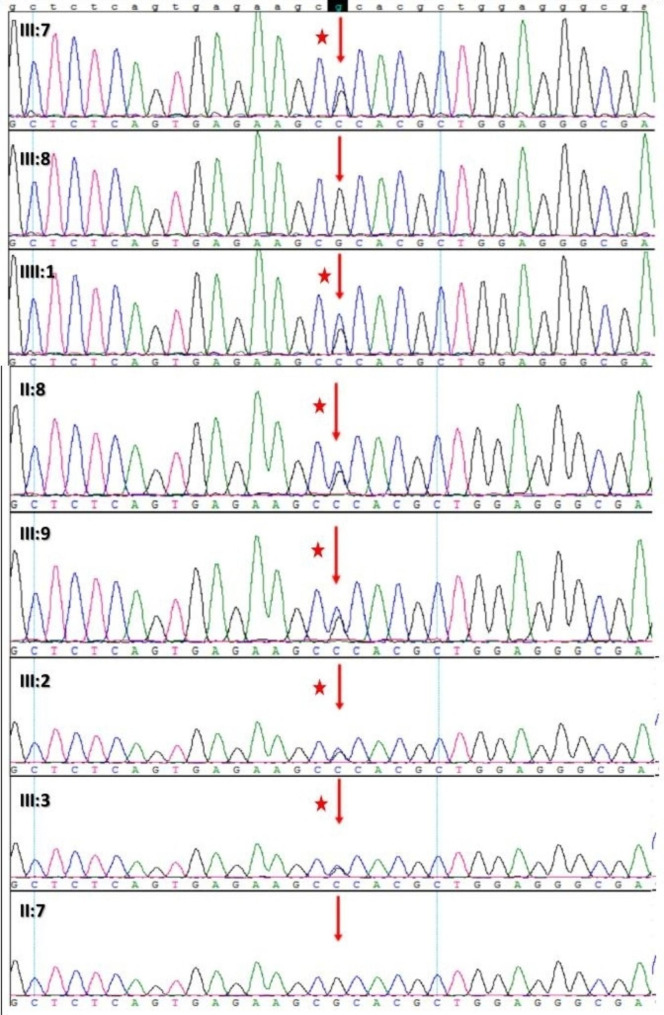
The verification of LMNA-c.467G > C(p.Arg156Pro) mutation by Sanger sequencing in family members. Red pentacle means carrying this heterozygous mutation

Compared with the mutant genes related to cardiovascular disease between OMIM database and the whole-exon sequencing results of the patients. We identified *LMNA* and *PRDM16* mutations as suspected pathogenic mutations for this family. Subsequently, we verified these two gene variants by Sanger sequencing in family members. Herein, we found that only *LMNA*(NM_170707.3):c.467G > C(p.Arg156Pro) was associated with the clinical phenotype (Fig. 4). The missense mutation of lamin A/C was not detected in the 1000 Genomes, ESP6500, ExAC, and gnomAD databases and was located in the conservative functional domain. This variant was predicted to be detrimental by bioinformatics protein function prediction software SIFT, MutationTaster, and Condel. According to the American College of Medical Genetics guidelines, the variant was identified as likely pathogenic (Evidence Level of Pathogenicity Assessment: PM1 + PM2 + PP1 + PP3) [24]. Also, the variant was not reported in the ClinVar database [25]. It is worth noting that, in this family, all the patients had ages of onset of DCM greater than 40 years of age. As p.Arg156Pro variant carrier, III:9 and IV:1 haven’t represented DCM manifestation, but the ongoing follow-up is necessary to them.

### Protein conformational analysis and prediction

The 3D visualization conformation of the target region is shown in Fig. 1B. Based on the protein structure prediction algorithm of AlphaFold2, the variation was located in the α-helix region structure of lamin A/C. Figure 1 C showed altered 156th amino acid that disrupted the arrangement of the hydrogen bonds and thus the α-helix structure.

### Mutation site of lamin A/C and statistical analysis

A total of 49 pathogenic amino acid mutation sites in lamin A/C were included in the statistical analysis. Of these, 24 were presented as cardiomyopathy, and 19/24 variations were located in the α-helix regions. Moreover, the structural modification occurred in the α-helix region accounting for 26/35 (74.3%) LMNA-related striated muscle diseases, including cardiomyopathy and musculoskeletal disorders. Supplementary Material 1 shows the clinical phenotype and clinical phenotype mutation location in the amino acid sequence of lamin A/C. We observed that the position of amino acid variation in some phenotypes exhibited an aggregation phenomenon. The variation of LMNA-related DCM was concentrated in the amino acid sequence 26–226, which formed an alpha helix chain structure, while the lipodystrophy-related variation was concentrated in the globular Ig-like domain (amino acids 427–547). To further verify whether the change in the alpha helix structure could cause DCM, we used the chi-square test to explore the relevance between protein structure and disease phenotype. As shown in Table 1, a statistical correlation was established between α-helix structural variation and DCM (p = 0.024, Pearson’s chi-square test). And in Table 2, the phenotype analysis of the striated muscle diseases (including cardiomyopathy and musculoskeletal disorders) revealed a statistically significant correlation between the α-helix structural variation and this phenotype (p = 0.011, Pearson’s chi-square test).

**Table 1:** The chi-square test of four-fold table between LMNA-DCM phenotype and α-Helix variation. The significance of association was defined by Pearson’s chi-square test

	LMNA-DCM	Non-LMNA-DCM	P-Value
α*-Helix variation*	19	12	
*Non-*α*-helix variation*	5	13	P = 0.024

**Table 2:** The chi-square test of four-fold table between Striated Muscle Disease phenotype and α-Helix variation. The significance of association was defined by Pearson’s chi-square test

	Striated Muscle Disease	Non- Striated Muscle Disease	P-Value
α*-Helix variation*	26	5	
*Non-*α*-helix variation*	9	9	P = 0.011

### Identification of DEGs in mice model and human heart tissue

**Fig. 5:**
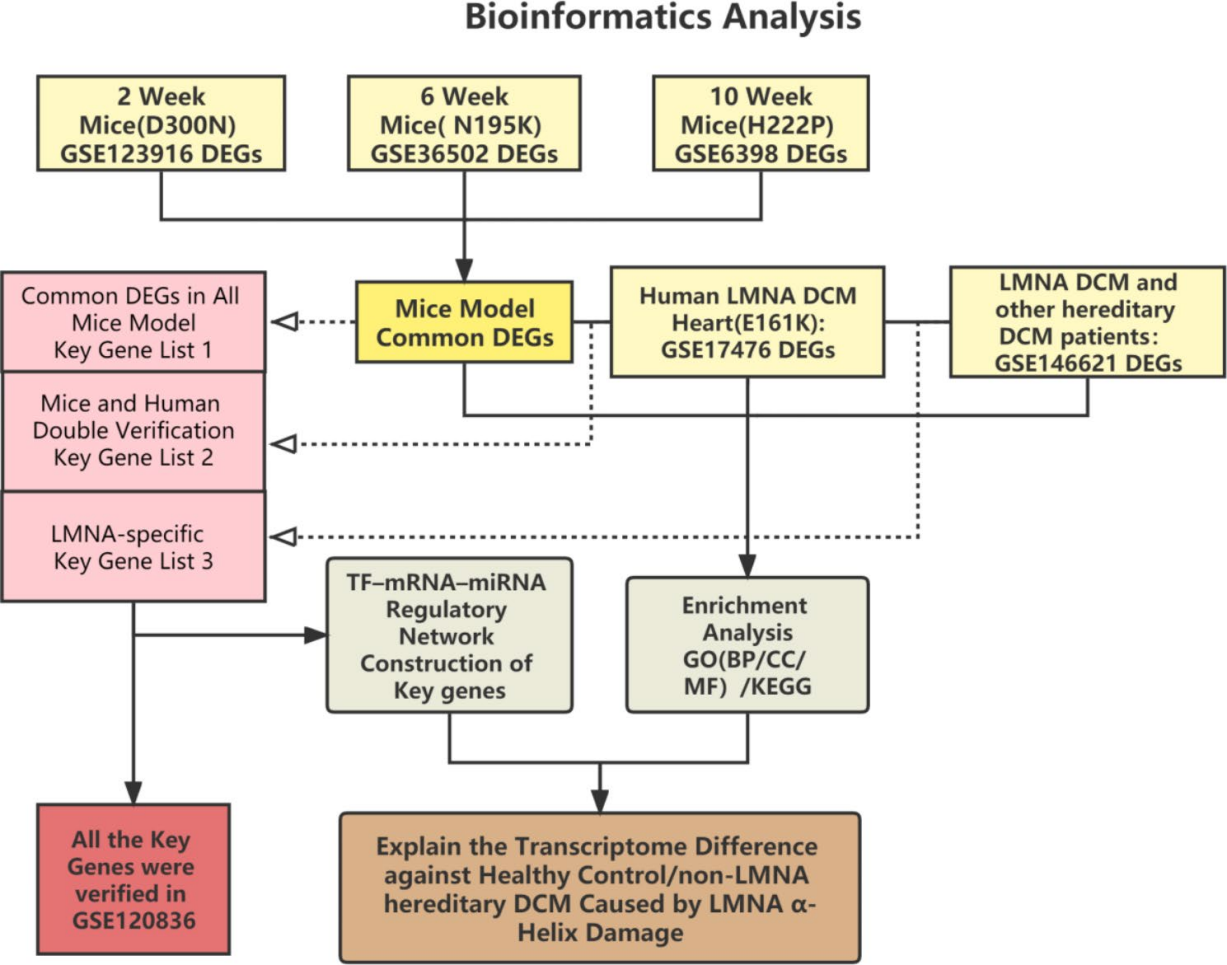
Bioinformatics Analysis design flowchart

The research design flow chart is illustrated in Fig. 5. Three cohorts of LMNA-DCM mice models and a human family of LMNA-DCM were evaluated in this study. All the mutations were detected in the α-helix region of lamin A/C. In 3 mice cohorts, 1078 DEGs were screened and 52 common DEGs were obtained by the intersection of the genes between at least two cohorts, only 3 DEGs (Key gene list 1) were actually overlapping with the 3 cohorts [Fig. 2A]. Moreover, we identified 394 upregulated and 278 downregulated LMNA-DCM DEGs. At the same time, we screened 119 LMNA-specific DEGs from LMNA-DCM patients and other hereditary DCM patients. As described in Method 2.7, we compared 3 kinds of DEGs, and 9 DEGs (Key gene list 2 and 3) were considered in subsequent studies.

### GO and KEGG pathway enrichment analyses of DEGs in LMNA-DCM

To decipher the biological roles of DEGs, the enrichment analysis was divided into four functional categories: biological processes (BP), cell component (CC), molecular function (MF), and KEGG pathway. The enrichment analysis of 52 mice cohorts common DEGs [Fig. 2 C] showed that the DEGs were specifically involved in the biological processes of positive regulation of cell migration (GO:0030335), regulation of hormone levels (GO:0010817), and positive regulation of cell motility (GO:2,000,147). In terms of the CC category, the mice model common DEGs were significantly involved in collagen trimer (GO:0005581), extracellular matrix (ECM) (GO:0031012), and external encapsulating structure (GO:0030312). For the MF category and KEGG pathway, the genes were mainly enriched in the signaling receptor regulator activity (GO:0030545), receptor-ligand activity (GO:0048018), PI3K-Akt signaling pathway (mmu04151), cell cycle (mmu04110), and MAPK signaling pathway (mmu04010). Moreover, enrichment analysis of human LMNA-DCM DEGs (between human patients and healthy controls) [Fig. 2D] indicated that the upregulated DEGs were mainly involved in the ECM organization (GO:0030198), collagen-containing extracellular matrix (GO:0062023), ECM structural constituent (GO:0005201), ECM-receptor interaction (hsa04512), focal adhesion (hsa04510), and PI3K-Akt signaling pathway (hsa04151). However, the analysis of the downregulated DEGs showed enrichment in cyclic-nucleotide-mediated signaling (GO:0019935), cytoplasmic ribonucleoprotein granule (GO:0036464), kinase binding (GO:0019900), oxytocin signaling pathway (hsa04921), cGMP-PKG signaling pathway (hsa04022), and gap junction (hsa04540).

In order to illustrate the transcriptomic changes from the α-helix variation in LMNA, we identified 119 special LMNA-DCM DEGs between LMNA-DCM and other hereditary DCM patients. In GO terms [Fig. 2E], these DEGs were mainly enriched in cell-cell adhesion (GO:0098609), regulation of leukocyte chemotaxis (GO:0002688), protein-DNA complex (GO:0032993), side of the membrane (GO:0098552), semaphorin receptor binding (GO:0030215), and chemorepellent activity (GO:0045499). In KEGG pathway terms, the LMNA-DCM DEGs were significantly enriched in neutrophil extracellular trap formation (hsa04613), natural killer cell-mediated cytotoxicity (hsa04650), and viral myocarditis (hsa05416).

### TF-mRNA-miRNA regulatory network construction and the validation of key genes

**Fig. 6:**
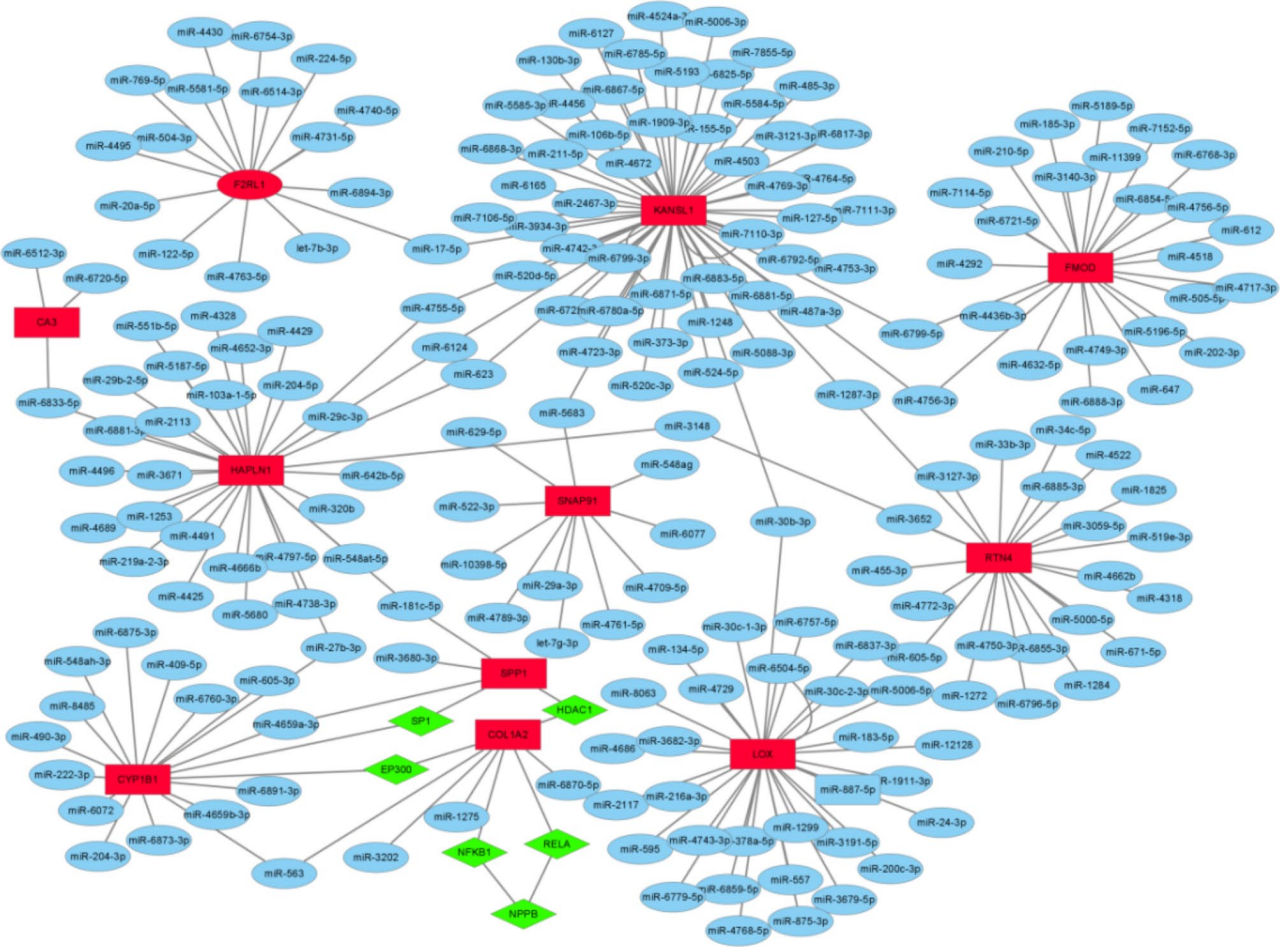
The TF–mRNA–miRNA regulation network of hub genes was constructed using the MiRwalk and TRRUST databases

**Fig. 7:**
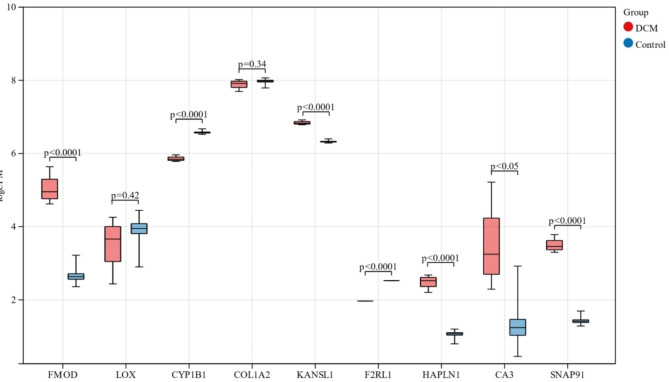
The expression levels of Key Genes of LMNA-DCM in GSE120836 (*P < 0.05; **P < 0.01). X-axis: Different Key Gene name. Y-axis: logCPM (Counts of exon model per Million mapped reads)

According to the established methods, we identified 12 genes (*CA3, LOX, SERPINA3N, SPP1, CYP1B1, FMOD, COLLA2, RTN4, KANSL1, SNAP91, HAPLN1*, and *F2RL1*) as key genes for an in-depth analysis in various datasets. Based on the MiRwalk and TRRUST database predictions of the key genes, the regulatory network of key genes and their predicted miRNAs and TFs was established and generated by using the Cytoscape software [Fig. 6]. Then, 206 miRNAs and 6 TFs [Supplementary Material 2] were obtained with the condition that their prediction could be verified by experiments or other databases. Based on the cross-linking of the network, we screened 15 miRNAs and 3 TFs (SP1, ESP300, and HDAC1), which might regulate the expression of the key genes. The expression of every key gene was verified in GSE120836, and 7 key genes (*FMOD*, *CYP1B1*, *KANSL1*, *F2RL1*, *HAPLN1*, *CA3*, and *SNAP91*) showed significant differences between LMNA-DCM and healthy controls [Fig. 7] (*SERPINA3N, SPP1* and *RTN4* were not detected in *GSE120836*).

## Discussion

In conventional genetic studies, the function of the pathogenic genes followed the gene-protein-disease routine. In the current study, we adopted the technology of whole-exome sequencing and combined the American College of Medical Genetics guidelines to identify pathogenic mutations in a DCM family. In the current study, we used AlphaFold2 to predict the structure of the mutated protein and successfully focused on the abnormal α-helix structure in this DCM family. The statistical analysis of phenotype-genotype data revealed the statistical significance of the destruction of α-helix in lamin A/C and LMNA-DCM. In the clinical study of hereditary diseases, the identification of pathogenic mutations and the interpretation of pathogenicity are crucial and challenging. The protein structure analysis bridges the gap between the mutated genes and disease phenotypes. In computational biology, AlphaFold2 software has ushered a revolution in high-quality protein structure prediction. We firstly applied it to genetic disease and explained the pathogenicity at the protein level. Owing to the convenience and accuracy of AlphaFold2, the 3D structure of the variant protein could be visualized easily, which provided supplemental significance to the research of genetic diseases and a new insight in the genetic diagnosis of inherited diseases.

The a-helix domain is a common secondary structure of proteins that widely participates in the formation of the cytoskeleton. Lamin A/C are the components of the nuclear lamina, a fibrous layer on the nucleoplasmic side of the inner nuclear membrane, which might provide a framework for the nuclear envelope and also interact with chromatin. α-helix domain is highly conserved and critical for the formation of the α-helical coiled-coil dimer, which is ascribed as the basic building block for the construction of lamin filaments. Laurini et al. studied three mutations of lamin A/C located in the central α-helical rod domain [26] and detected low resistance to deformation and damaged transmission of mechanical stresses from the membrane to the nucleus due to a defective actin structure. Lamins are critical filament proteins which facilitate the cells to resist mechanical stress and generate force. They are connected to the cytoskeleton via the linker of the nucleoskeleton and cytoskeleton (LINC) complex. Thus, the LINC complex could be the potential therapeutic target for LMNA-DCM. In muscle cells, the expression of the p.H222P in lamin A/C increases cellular stiffness; the chemical agent selumetinib, an inhibitor of the ERK1/2 signaling, could reverse the mechanical alterations in the mutated cells [27]. A similar event occurred in myocardial cells in a specific LMNA deletion mice model, which damaged the LINC complex structure caused by knockout or knockdown of *SUN1* (a LINC complex protein gene) and in turn, prevented cardiomyopathy progression and extended the lifespan [28].

To better illustrate the effect of α-helical variation on the heart, we downloaded the transcriptome data of LMNA-DCM mice model and patient, screened multiple groups of DEGs, and identified 7 key genes in LMNA-DCM. Functional annotation analysis revealed that different groups of DEGs are commonly involved in collagen formation and ECM. The commonly enriched PI3K-Akt signaling pathway and MAPK signaling pathway in the human and mice model datasets were valuable in revealing the pathogenic mechanism of α-helical variation in the *LMNA* gene from the biological perspective. Similar to our enrichment results, Cai et al. established an LMNA R225X knock-in mice model and discovered ECM remodeling and accumulation of collagen in the heart [29]. Metabolomics analysis also confirmed the abnormal collagen degradation in LMNA-DCM heart [30]. In the cTnT^R141W^ transgenic DCM mice, 24-dehydrocholesterol reductase activates the PI3K-Akt pathway and protects against DCM [31]. Cattin et al. pointed out that the MAPK and PI3K-Akt signaling pathways are activated early in LMNA-DCM, and inhibition of these pathways was beneficial to the cardiac tissue [32]. We also speculated that the MAPK and PI3K-Akt pathways could be the potential treatment targets in LMNA-DCM.

Subsequently, an integrated approach was adopted to identify the vital genes in the progression of LMNA-DCM. Combined with the results of the validation dataset, the differential expression of 7 key genes was confirmed in LMNA-DCM. Among these, *FMOD*, *CYP1B1*, and *CA3* showed a characteristic expression in LMNA-DCM compared to the healthy heart. *FMOD* belongs to the family of small interstitial proteoglycans and plays a role in ECM assembly. Its biological function coincides with the critical pathway enriched previously. Moreover, *FMOD* could sequester the transforming growth factor-beta (TGF-β) into the ECM to regulate its activities that are closely related to cardiac remodeling and fibrosis [33]. Meta-analysis and co-expression analysis commonly verified the differential expression of *FMOD* in DCM patients and healthy individuals [34]. This differential expression was also observed in hypertrophic cardiomyopathy [35]. *CYP1B1* is one of the cytochrome P450 enzymes associated with arachidonic acid (AA) metabolites. CYP1B1 can metabolize AA into cardiotoxic metabolites exemplified as mid-chain hydroxyeicosatetraenoic acid (HETE), and 20-HETE is involved in the pathogenesis of cardiac hypertrophy and the consequent cardiac remodeling [36, 37]. As the inhibitor of CYP1B1, resveratrol attenuated cardiomyocyte hypertrophy through cardiotoxic mid-chain HETE metabolites [38]. In the study by Lu et al., the elevation of CYP1B1 transcript was observed in both DCM and ischemic cardiomyopathy heart tissues [39]. As a carbonic anhydrase enzyme, CA3 catalyzes the reversible hydration of carbon dioxide to bicarbonate in mammalian cells. It is also abundant in the heart and involved in cardiomyocyte energy metabolism. Similarly, recent studies have reported the function of CA3 in DCM research. Su et al. demonstrated that the expression of CA3 is remarkably higher in the plasma of the DCM patient [40]. Feng et al. constructed CA3-knockout mice models and demonstrated the cardioprotective effect of CA3 against acidotic stress [41]. In the in vitro experiments, overexpressed CA3 exerted an inhibitory effect on hypoxia-induced cardiomyocyte apoptosis [42]. Thus, we speculated that the increase in CA3 is a vital function of self-protection in DCM. Therefore, CA3 could be a risk biomarker, while appropriately increasing the expression of CA3 could be a therapeutic target in LMNA-DCM.

Notably, the above three key genes were identified from the comparison between LMNA-DCM and healthy controls. Strikingly, these genes could not independently reflect the transcriptomic differences caused by LMNA gene mutation. Hence, we conducted a differential gene analysis between LMNA-DCM heart and other genetic mutations in DCM and identified 4 key genes (*KANSL1*, *F2RL1*, *HAPLN1*, and *SNAP91*). *KANSL1* is an essential gene for autophagy, which has been confirmed to be related to damaged autophagic clearance and accumulation of reactive oxygen species, thereby resulting in defective cardiac functions [43]. In 22q11.2 microdeletion syndrome patients, *KANSL1* was also reported to be associated with an increased risk of congenital heart defects [44]. *F2RL1* is also known as protease-activated receptor 2 (*PAR2*) and is widely expressed in the cardiovascular system and plays critical roles in immune cells, fibroblasts, and cardiomyocyte function. In heart failure with preserved ejection fraction patients, low myocardial *PAR2* gene expression is associated with aggravated diastolic dysfunction and ECM remodeling [45]. However, in myocardial infarction [46] and atherosclerosis [47] models, the expression of *PAR2* exhibits a pro-fibrotic, pro-inflammatory phenotype. In the current study, we observed the expression of *PAR2* was specifically decreased in LMNA-DCM. This controversial phenomenon enlightened a new pathological mechanism of LMNA-DCM and needs further exploration. *HAPLN1* encoding hyaluronan and proteoglycan link protein which maintains stable aggregation of vital ECM macromolecules [48]. *HAPLN1* is also involved in the proliferation and activation of fibroblasts in lung tissue [49], synovial tissue [50], and tumor tissue [51]. Thus, we could speculate *HAPLN1* plays a similar role in cardiac fibroblasts and promotes fibrosis and matrix remodeling in LMNA-DCM. Synaptosome-associated protein 91 is encoded by *SNAP91* and acts upstream of or within the regulation of clathrin-dependent endocytosis. In the GO annotations, *SNAP91* participates in protein kinase binding and clathrin binding. Different from other key genes, only a few studies concentrated on the function of *SNAP91*. Firstly, we observed the high expression of *SNAP91* in LMNA-DCM; however, the specific molecular mechanism is yet to be elucidated.

In the current study, we started with the phenotype and explored the statistical significance between site-specific mutation and specific phenotype. The structure prediction by Alphafold2 helped us to interpret mutation at the protein level, revealed the function of different protein domains, and proposed new therapeutic targets. This method is especially applicable for multifunctional and multi-pathogenic genes. With the promotion of sequencing technology and the accumulation of pathogenic mutations, this phenotype-genotype reverse mode could provide new ideas for understanding genetic diseases. Moreover, by analyzing the α-helix targeting variation transcriptomics data in LMNA-DCM, we comprehensively expounded the biological change at the transcriptome level. Thus, the critical biological pathway and the key genes could be the biomarkers and potential therapeutic targets for future research.

Nevertheless, there were still some deficiencies worthy to be mentioned in this study. First of all, the LMNA-DCM was not a common genetic disorder, we couldn’t get enough patients for further research. Although we added transcriptomic data of animal models for multi-angle screening and validation. But the animal models were observed at different ages (2, 4 and 10 weeks). Because gene expression is directly related to development, it is possible that comparing animal cohorts with different ages highlights developmental genes in addition to genes associated to LMNA mutation. We hope to accumulate more clinical cases and perform the certain age of animal models to do more in-depth research in the future.

## Conclusion

The novel variant in *LMNA*:c.467G > C(p.Arg156Pro) exhibited an interrupted α-helix region and resulted in cardiomyopathy manifestations. The α-helical variation is related to the intermediate filaments binding and could be responsible for LMNA-DCM. Bioinformatics analysis provided new insights into the distinct mechanisms of this structural variation, and the key genes screened in this study deciphered the potential biomarkers or therapeutic targets in LMNA-DCM.

### Electronic supplementary material

Below is the link to the electronic supplementary material.

Supplementary Material 1

## Data Availability

The microarry and RNA-seq datasets in this study are available from the Gene Expression Omnibus (GEO, http://www.ncbi.nlm.nih.gov/geo/). The accession number of data series are GSE123916, GSE36502, GSE6398, GSE17476, GSE146621 and GSE120836.

## Abbreviations

LMNA-DCM: LMNA-related dilated cardiomyopathy
OMIM: Database Online Mendelian Inheritance in Man database
DEGs: Differentially expressed genes
TF: Transcription factors
EDMD: Emery-Dreifuss muscular dystrophy
HGPS: Hutchinson-Gilford progeria syndrome
SCD: Sudden cardiac death
LVEF: Left ventricular ejection fraction
ICD: Implantable cardioverter-defibrillator
PCR: Polymerase chain reaction
GEO: Database Gene Expression Omnibus database
GO: Gene Ontology
KEGG: Kyoto Encyclopedia of Genes and Genomes
LVNC: Left ventricular noncompaction
CRT-D: Cardiac resynchronization therapy with defibrillator
BP: Biological process
CC: Cell component
MF: Molecular function
ECM: Extracellular matrix
LINC: Linker of the nucleoskeleton and cytoskeleton
