# Supplementary material for: An alpha-helix variant p.Arg156Pro in LMNA as a cause of hereditary dilated cardiomyopathy: genetics and bioinfomatics exploration
Source: BMC Med Genomics. 2023 Oct 2;16:229. doi: 10.1186/s12920-023-01661-1 (PMC10544607; doi:10.1186/s12920-023-01661-1)
Supplement: Supplementary file 1 — Supplementary Material 1 [file 12920_2023_1661_MOESM1_ESM.docx]

| **Num** | **Phenotype** | **AA site** | **Mutation** | **source** | **Structure** |
| --- | --- | --- | --- | --- | --- |
| 1 | Cardiomyopathy | 57 | ALA57PRO | OMIM Database | α-helix 1 (Amino Acid 26-226) |
| 2 | Cardiomyopathy | 59 | LEU59ARG | OMIM Database |  |
| 3 | Cardiomyopathy;Hyperlipidemia | 60 | ARG60GLY | OMIM Database |  |
| 4 | Cardiomyopathy | 62 | ARG62GLY | PMID:20041886[1] |  |
| 5 | Cardiomyopathy | 85 | LEU85ARG | OMIM Database |  |
| 6 | Cardiomyopathy | 89 | ARG89LEU | PMID:20160190[2] |  |
| 7 | Cardiomyopathy | 101 | ARG101PRO | PMID:20160190[2] |  |
| 8 | Hyperlipidemia;HGPS;Musculoskeletal Disease | 133 | ARG133LEU ARG133PRO | OMIM Database |  |
| 9 | HGPS | 140 | LEU140ARG | OMIM Database |  |
| 10 | Musculoskeletal Disease | 143 | SER143PHE | OMIM Database |  |
| 11 | HGPS | 145 | GLU145LYS | OMIM Database |  |
| 12* | Cardiomyopathy | 156 | ARG156PRO | New findings* |  |
| 13 | Cardiomyopathy | 161 | GLU161LYS | OMIM Database |  |
| 14 | Cardiomyopathy | 166 | ARG166PRO | PMID:20160190[2] |  |
| 15 | Cardiomyopathy | 190 | ARG190QLN | PMID:20160190[2]、18795223[3] |  |
| 16 | Cardiomyopathy | 192 | ASP192GLY | PMID:29432544 |  |
| 17 | Cardiomyopathy | 195 | ASN195LYS | OMIM Database |  |
| 18 | Cardiomyopathy | 203 | GLU203GLY GLU203LYS | OMIM Database |  |
| 19 | Cardiomyopathy | 219 | LYS219THR | PMID:18795223[3] |  |
| 20 | Cardiomyopathy | 210 | ILE210SER | PMID:20160190[2] |  |
| 21 | Cardiomyopathy | 215 | LEU215PRO | PMID:20160190[2] |  |
| 22 | Musculoskeletal Disease | 222 | HIS222TYR | OMIM Database |  |
| 23 | Musculoskeletal Disease | 225 | ARG225GLN | OMIM Database |  |
| 24 | Hyperlipidemia | 230 | ASP230ASN | OMIM Database | Single Chain (Amino Acid 227-231) |
| 25 | Musculoskeletal Disease | 249 | ARG249TRP | OMIM Database | α-helix 2 (Amino Acid 231-386) |
| 26 | Musculoskeletal Disease | 298 | ARG298CYS | OMIM Database |  |
| 27 | HGPS | 300 | ASP300GLY | OMIM Database |  |
| 28 | Heart-Hand Syndrome | 335 | ARG335TRP | OMIM Database |  |
| 29 | Cardiomyopathy | 347 | GLU347LYS | PMID:22266370[4] |  |
| 30 | Musculoskeletal Disease | 358 | GLU358LYS | OMIM Database |  |
| 31 | Cardiomyopathy;Musculoskeletal Disease | 377 | ARG377HIS | PMID:12920062[5] |  |
| 32 | Musculoskeletal Disease | 380 | LEU380SER | OMIM Database |  |
| 33 | Cardiomyopathy | 388 | ARG388HIS | PMID:20160190[2] | Single Chain (Amino Acid 387-426) |
| 34 | Hyperlipidemia | 399 | ARG399CYS | OMIM Database |  |
| 35 | Mandibuloacral Dysplasia;Hyperlipidemia | 440 | VAL440MET | OMIM Database | Globular Immunoglobulin (Ig)-like domain (Amino Acid 427-547) |
| 36 | Musculoskeletal Disease | 453 | ARG453TRP | OMIM Database |  |
| 37 | Hyperlipidemia | 465 | GLY465ASP | OMIM Database |  |
| 38 | Mandibuloacral Dysplasia;Hyperlipidemia | 471 | ARG471CYS | OMIM Database |  |
| 39 | Hyperlipidemia;Musculoskeletal Disease | 482 | ARG482GLN ARG482TRP ARG482LEU | OMIM Database |  |
| 40 | Musculoskeletal Disease;Hyperlipidemia | 527 | ARG527PRO ARG527HIS ARG527CYS | OMIM Database |  |
| 41 | Mandibuloacral Dysplasia;Hyperlipidemia | 529 | ALA529VAL ALA529THR | OMIM Database |  |
| 42 | Musculoskeletal Disease | 530 | LEU530PRO | OMIM Database |  |
| 43 | Cardiomyopathy | 541 | ARG541GLY | OMIM Database |  |
| 44 | Mandibuloacral Dysplasia;Hyperlipidemia | 542 | LYS542ASN | OMIM Database |  |
| 45 | Cardiomyopathy | 571 | ARG571SER | OMIM Database | Single Chain (Amino Acid 548-661) |
| 46 | Cardiomyopathy;Mandibuloacral Dysplasia;Hyperlipidemia | 573 | SER573LEU | OMIM Database |  |
| 47 | Hyperlipidemia | 582 | ARG582HIS | OMIM Database |  |
| 48 | HGPS | 608 | GLY608SER | OMIM Database |  |
| 49 | Cardiomyopathy | 644 | ARG644CYS | PMID:18795223[3] |  |

**Supplementary material 1.** All of the [pathogenic](javascript:;) missense mutations of LMNA which was included in OMIM database or reported in DCM family. The correspondence of phenotype information, amino acid variation sites and the structures were reflected in the table.

.

| **MicroRNAs in TF–mRNA–miRNA Regulatory Network** |
| --- |
| let-7b-3p let-7g-3p miR-10398-5p miR-103a-1-5p miR-106b-5p miR-11399  miR-12128 miR-122-5p miR-1248 miR-1253 miR-1272 miR-1275 miR-127-5p  miR-1284 **miR-1287-3p** miR-1299 miR-130b-3p miR-134-5p miR-155-5p **miR-17-5p** **miR-181c-5p** miR-1825 miR-183-5p miR-185-3p miR-1909-3p miR-1911-3p  miR-200c-3p miR-202-3p miR-204-3p miR-204-5p miR-20a-5p miR-210-5p  miR-2113 miR-211-5p miR-2117 miR-216a-3p miR-219a-2-3p miR-222-3p  miR-224-5p miR-24-3p miR-2467-3p **miR-27b-3p** miR-29a-3p miR-29b-2-5p  miR-29c-3p miR-3059-5p **miR-30b-3p** miR-30c-1-3p miR-30c-2-3p miR-3121-3p miR-3127-3p miR-3140-3p **miR-3148** miR-3191-5p miR-3202 miR-320b miR-33b-3p miR-34c-5p miR-3652 miR-3671 miR-3679-5p miR-3680-3p miR-3682-3p  miR-373-3p miR-378a-5p miR-3934-3p miR-409-5p miR-4292 miR-4318 miR-4328 miR-4425 miR-4429 miR-4430 miR-4436b-3p miR-4456 miR-4491 miR-4495  miR-4496 miR-4503 miR-4518 miR-4522 miR-4524a-3p miR-455-3p miR-4632-5p miR-4652-3p **miR-4659a-3p** miR-4659b-3p miR-4662b miR-4666b miR-4672  miR-4686 miR-4689 miR-4709-5p miR-4717-3p miR-4723-3p miR-4729 miR-4731-5p miR-4738-3p miR-4740-5p miR-4742-3p miR-4743-3p miR-4749-3p miR-4750-3p miR-4753-3p **miR-4755-5p miR-4756-3p** miR-4756-5p miR-4761-5p miR-4763-5p miR-4764-5p miR-4768-5p miR-4769-3p miR-4772-3p miR-4789-3p miR-4797-5p miR-485-3p miR-487a-3p miR-490-3p miR-5000-5p miR-5006-3p miR-5006-5p  miR-504-3p miR-505-5p miR-5088-3p miR-5187-5p miR-5189-5p miR-5193  miR-5196-5p miR-519e-3p miR-520c-3p miR-520d-5p miR-522-3p miR-524-5p  miR-548ag miR-548ah-3p miR-548at-5p miR-551b-5p miR-557 miR-5581-5p  miR-5584-5p miR-5585-3p **miR-563** miR-5680 **miR-5683** miR-595 miR-605-3p  miR-605-5p miR-6072 miR-6077 miR-612 **miR-6124** miR-6127 miR-6165 **miR-623** miR-629-5p miR-642b-5p miR-647 miR-6504-5p miR-6512-3p miR-6514-3p  miR-671-5p miR-6720-5p miR-6721-5p miR-6722-3p miR-6754-3p miR-6757-5p miR-6760-3p miR-6768-3p iR-6780a-5p miR-6785-5p miR-6792-5p miR-6796-5p miR-6799-3p **miR-6799-5p** miR-6817-3p miR-6825-5p **miR-6833-5p** miR-6837-3p miR-6854-5p miR-6855-3p miR-6859-5p miR-6867-5p miR-6868-3p miR-6870-5p miR-6871-5p miR-6873-3p miR-6875-3p miR-6881-3p miR-6881-5p miR-6883-5p miR-6885-3p miR-6888-3p miR-6891-3p miR-6894-3p miR-7106-5p miR-7110-3p miR-7111-3p miR-7114-5p miR-7152-5p miR-769-5p miR-7855-5p miR-8063  miR-8485 miR-875-3p miR-887-5p |
| **Transcription Factors in TF–mRNA–miRNA Regulatory Network** |
| **SP1 HDAC1 EP300** NFKB1 RELA NPPB |

**Supplementary material 2.** All of the microRNAs and TFs in TF–mRNA–miRNA Regulatory Network. The yellow markers highlight the hub microRNA and TF which regulated multiple key genes.

**References:**

1. Subramanyam L, Simha V, Garg A. Overlapping syndrome with familial partial lipodystrophy, Dunnigan variety and cardiomyopathy due to amino-terminal heterozygous missense lamin A/C mutations. *CLIN GENET* 2010; 78(1):66-73.

2. Cowan J, Li D, Gonzalez-Quintana J, Morales A, Hershberger RE. Morphological analysis of 13 LMNA variants identified in a cohort of 324 unrelated patients with idiopathic or familial dilated cardiomyopathy. *Circ Cardiovasc Genet* 2010; 3(1):6-14.

3. Perrot A, Hussein S, Ruppert V, Schmidt HH, Wehnert MS, Duong NT, Posch MG, Panek A, Dietz R, Kindermann I, Bohm M, Michalewska-Wludarczyk A, Richter A, Maisch B, Pankuweit S, Ozcelik C. Identification of mutational hot spots in LMNA encoding lamin A/C in patients with familial dilated cardiomyopathy. *BASIC RES CARDIOL* 2009; 104(1):90-99.

4. Bollati M, Barbiroli A, Favalli V, Arbustini E, Charron P, Bolognesi M. Structures of the lamin A/C R335W and E347K mutants: implications for dilated cardiolaminopathies. *Biochem Biophys Res Commun* 2012; 418(2):217-221.

5. Sebillon P, Bouchier C, Bidot LD, Bonne G, Ahamed K, Charron P, Drouin-Garraud V, Millaire A, Desrumeaux G, Benaiche A, Charniot JC, Schwartz K, Villard E, Komajda M. Expanding the phenotype of LMNA mutations in dilated cardiomyopathy and functional consequences of these mutations. *J MED GENET* 2003; 40(8):560-567.
